# The Leaf Trichome, Venation, and Mesophyll Structural Traits Play Important Roles in the Physiological Responses of Oak Seedlings to Water-Deficit Stress

**DOI:** 10.3390/ijms23158640

**Published:** 2022-08-03

**Authors:** Jonathan O. Hernandez, Byung Bae Park

**Affiliations:** 1 Department of Forest Biological Sciences, College of Forestry and Natural Resources, University of the Philippines Los Baños, Laguna 4031, Philippines; johernandez2@up.edu.ph; 2 Department of Environment and Forest Resources, College of Agriculture and Life Science, Chungnam National University, Daejeon 34134, Korea

**Keywords:** drought stress, drought tolerance, oak species, starch reserves, stomatal conductance, water use efficiency

## Abstract

In this study, we investigated the effects of water-deficit stress on the leaf anatomical traits, physiological traits, and stem starch content in *Quercus acutissima* Carruth and *Quercus serrata* Murray by subjecting their seedlings to well-watered (WW) and water-deficit stress (WS) treatments. The water stress-induced changes in trichome density, trichome-to-stomata ratio, mesophyll thickness, vein density, vein distance, vein loopiness, vessel diameter, transpiration (E), stomatal conductance (g_s_), water use efficiency (WUE), and starch content were analyzed between two time points. While trichome density did not vary between treatments in *Q. acutissima*, it dramatically increased in *Q. serrata* (62.63–98.96 trichomes mm^−2^) at the final week. The WS-treated seedlings had a thicker palisade mesophyll (162.85–169.56 µm) than the WW-treated samples (118.56–132.25 µm) in both species. The vein density and loopiness increased significantly in the WS-treated *Q. serrata* seedlings. Small-sized vessels (10–50 µm) were more frequent in the WS than the WW in *Q. serrata*. The E, g_s_, WUE, and starch content declined significantly in the WS-treated seedlings compared with WW-treated samples in both species. Further, principal component analysis revealed significant relationships between anatomical and physiological traits, particularly in the WS-treated seedlings of *Q. serrata*. The coordinated changes in leaf anatomical traits, physiological traits, and stem starch content indicate an important role in the survival of *Q. acutissima* and *Q. serrata* seedlings in water-deficit stress environments, although *Q. serrata* may show higher survivability under prolonged water stress than *Q. acutissima*.

## 1. Introduction

Drought occurrence has risen by 29% since 2000, and more than 75% of the world could experience drought by 2050 [1]. Drought has been identified as a major factor in reducing tree growth and forest productivity by altering forest structure and soil–root and leaf–atmosphere interactions [2,3]. These alterations are likely to happen often at larger spatiotemporal scales because of worsening global warming and the unpredictable effects of climate change [4,5]. Consequently, identifying which tree species are more vulnerable or resistant to water-deficit stress is important for managing our forests amid climate change. The increasing frequency of drought has engendered proactive approaches and strong commitments to drought preparedness and resilience, including the use of drought-resistant and fast-growing species, in the reforestation programs in many countries.

Some plants exhibit morpho-anatomical and physiological mechanisms to respond effectively to water-deficit stress [6]. Morphologically, leaves with dense trichomes per unit of leaf area are less susceptible to abiotic damage by limiting excessive water loss using transpiration and the regulation of leaf temperature [7]. Trichomes can be non-glandular or glandular and can be found on approximately 30% of all vascular plant species [8]. Both types can secrete or store large quantities of specialized metabolites, which help improve plant fitness to adverse environmental conditions [9]. A study found that trichome density was significantly higher in the dry season than in the wet season, influencing the physiological activities of plants [10]. The presence of leaf trichomes can directly influence various physiological processes, including transpiration rate, stomatal conductance, and water use efficiency [11]. In *Solanum* species, the ratio of trichomes to stomata was positively correlated with WUE [12]. Woolly trichome mutants showed a higher stomatal conductance than plants without trichomes [13]. While this information is well-documented in many herbaceous crops, the relationship between trichome density and physiological traits remains unclear under water-deficit stress in many forest tree species. A better understanding of such a relationship will help elucidate the increasing vulnerability of forest tree species to drought.

The effects of drought on mesophyll, venation, and biochemical traits of plants also remain unclear. These traits are some of the limiting processes that control photosynthetic capacity and water use efficiency of plants under water-deficit stress conditions [14]. The leaves of drought-adapted plants typically have more closely-pack, elongated cells, but with thinner spongy mesophyll cells (higher palisade to spongy ratio) than those of drought-susceptible plants [15]. A previous study found a significant difference in the number of palisade cells between drought-treated (i.e., 40–50 cells) and control plants (i.e., 29–36 cells) [16]. A similar study also reported thicker palisade tissue of drought-tolerant plants compared with drought-sensitive plants of *Brassica napus* L. [17]. Structural damage in palisade cells caused by drought stress resulted in the depletion of starch reserves [18]. Damage to the architecture of the leaf veins also affects the efficiency of the gas exchange in the leaf and thus, the production of starch reserves [19,20]. Water deficiency can shrivel the leaves, thus leading to serious structural damage to the vein networks, mesophyll tissues, and plastids, where starch is synthesized [21,22]. Previous studies demonstrated that starch reserves play a key role in maintaining growth and sustaining the energy of plants under drought stress [23]. During the photoperiod, starch reserves in plants are increased under water stress conditions and are converted into soluble sugars as osmolytes to support plant growth [24]. The effects of drought on starch reserves is relatively well-documented in many herbaceous plants, but the potential influence of structural damage to palisade cells on starch reserves as the leaves shrivel needs further investigation, particularly in long-vessel angiosperms, such as oaks.

Previous studies suggest that oak trees and other ring-porous species in temperate and tropical zones are more susceptible to drought than other tree species because of their longer and wider water-conducting vessels, which are prone to conduction-blocking embolisms [25,26]. Thus, further studies on how oak species respond to water-deficit stress are imperative for predicting their survival amid increasing drought severity in the future. In this study, we investigated the effects of water-deficit stress on the leaf anatomical traits, physiological traits, and stem starch content in oak seedlings. We hypothesized that in order for the water deficit stress-treated seedlings to survive progressive water stress and minimize the negative effects on the transpiration rate, stomatal conductance, and water use efficiency, they would need to exhibit more pronounced changes in trichome density, trichome-to-stomata ratio, venation, mesophyll thickness, xylem vessels diameter, and starch content compared with the well-watered seedlings. A quantitative understanding of these responses at the anatomical/cellular level is fundamental in predicting how tree vegetation will respond to future climate changes.

## 2. Results

### 2.1. Leaf Anatomical Structures in Well-Watered and Water Deficit Stressed Seedlings

**Figure 1:**
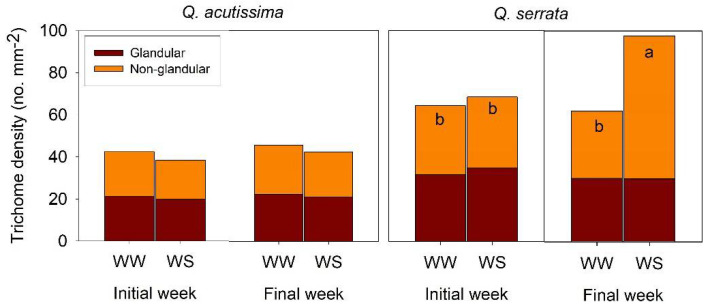
Density of glandular and non-glandular trichomes of *Quercus acutissima* and *Q. serrata* in the well-watered (WW) and water-deficit stress (WS) treatments. Comparison means derived using Tukey’s test (*p* < 0.05) are shown for the significant interaction between water stress and time. Different lowercase letters indicate significant differences (n = 10). Still-green leaves were the ones harvested for leaf anatomical traits measurement.

**Figure 2:**
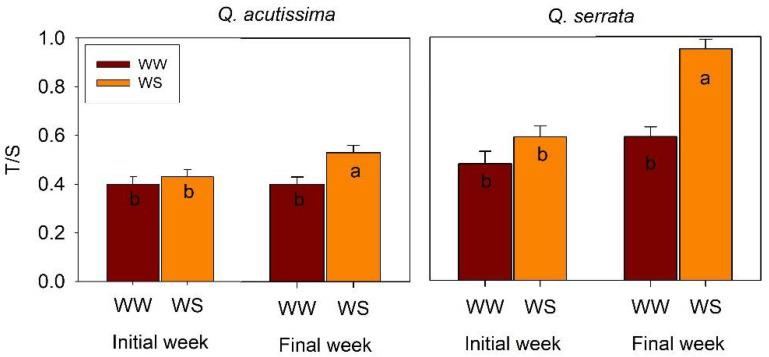
Trichome-to-stomata ratio (T/S) of *Quercus acutissima* and *Q. serrata* in well-watered (WW) and water-deficit stress (WS) treatments. Vertical bars indicate the SE (n = 10). Comparison means derived using Tukey’s test (*p* < 0.05) are shown for the significant interaction between water stress and time. Different lowercase letters indicate significant differences.

**Figure 3:**
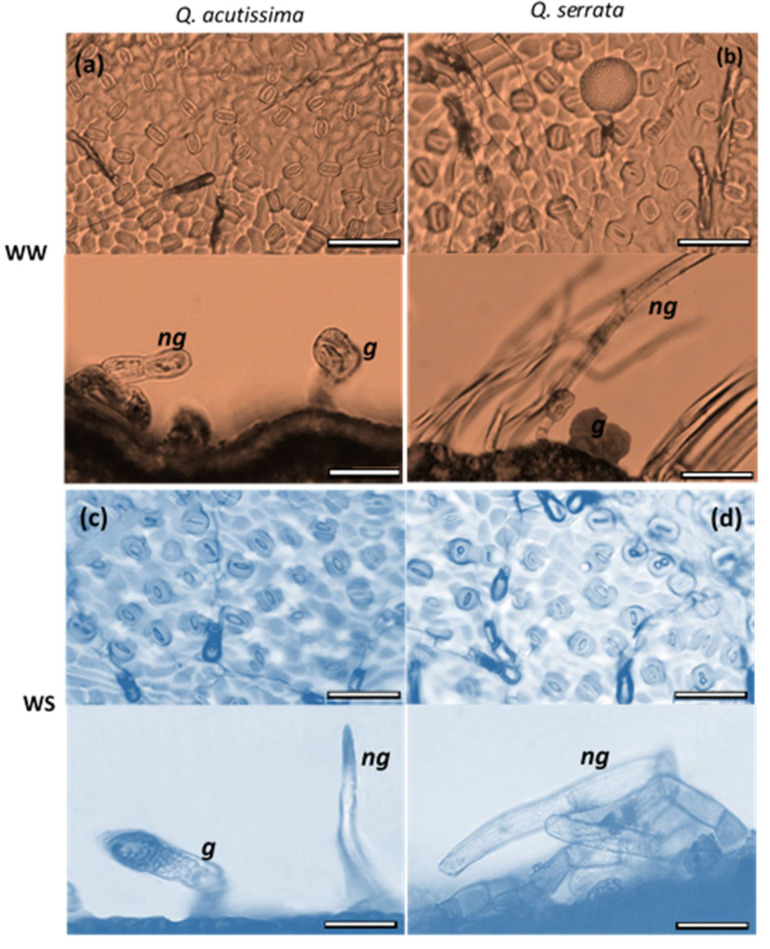
Glandular (g) and non-glandular trichomes (ng) of *Quercus acutissima* and *Q. serrata* in (**a**,**b**) well-watered (WW) and (**c**,**d**) water-deficit stress (WS) treatments at the final week. The bar represents 59. 5 µm.

Two types of abaxial surface trichomes were observed on both WW and WS seedlings of *Q. acutissima* and *Q. serrata* (Figure 1). Trichome density of all types (glandular and non-glandular) was similar between the WW and WS treatments in both species at the initial week, but a significant variation (*p* < 0.001) was detected at the final week in the *Q. serrata* seedlings (Figure 1). The density of trichomes in *Q. serrata* significantly increased from 62.63 trichomes mm^−2^ in WW to 98.96 trichomes mm^−2^ in WS. Specifically, the density of the non-glandular trichomes increased in the WS-treated seedlings of *Q. serrata* compared with the WW-treated seedlings (Figure 1, Figure 2 and Figure 3). Moreover, we observed a substantially (*p* < 0.001) higher T/S in WS than WW in *Q. serrata* and *Q. acutissima* at the final week (Figure 2 and Figure 3).

**Figure 4:**
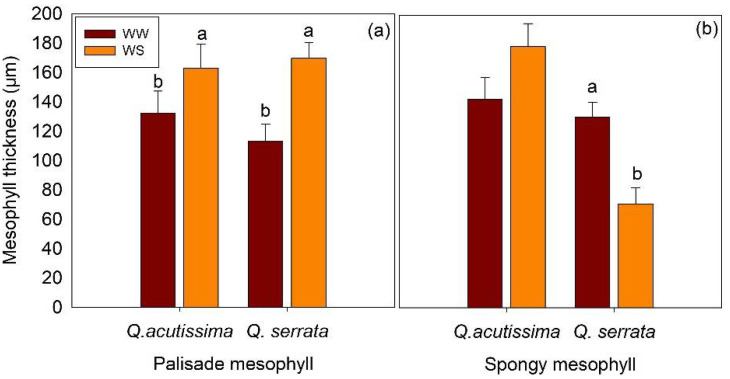
(**a**) Thickness of palisade mesophyll and (**b**) spongy mesophyll tissues of *Quercus acutissima* and *Q. serrata* in well-watered (WW) and water-deficit stress (WS) treatments. The vertical bars indicate the SE (n = 10). Different lowercase letters indicate significant differences between treatments at α = 0.05. Still-green leaves were the ones harvested for leaf anatomical traits measurement.

**Figure 5:**
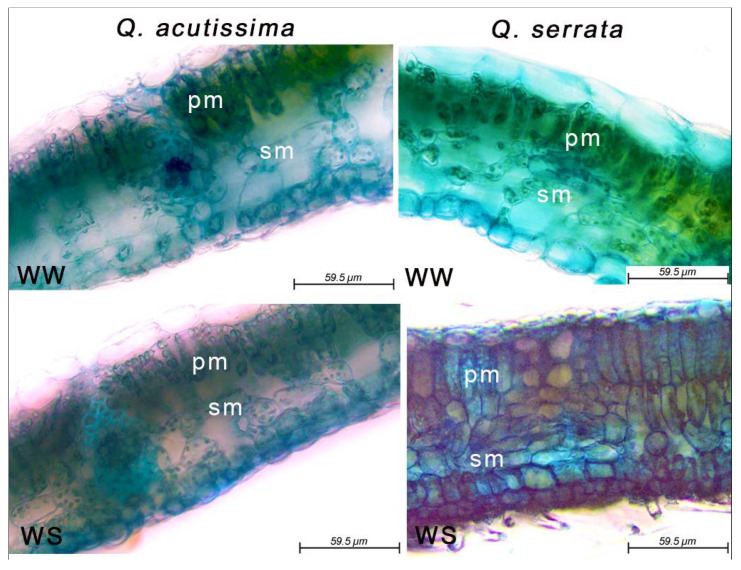
Changes in leaf anatomical structure of *Quercus acutissima* and *Q. serrata* showing the palisade mesophyll (pm) and spongy mesophyll (sm) tissues in well-watered (WW) and water-deficit stress (WS) treatments at the final week.

The thickness of palisade and spongy mesophyll tissues of the two species varied significantly (*p* < 0.05) between the WW and WS treatments (Figure 4). Here, the WS-treaded seedlings had thicker palisade mesophyll (162.85–169.56 µm) than the WW-treated samples (118.56–132.25 µm) in both *Q. acutissima* and *Q. serrata*. The mesophyll cells of the leaves of the control plants had individual parenchyma cells that were larger and looser than those in the water-stressed plants, especially in *Q. serrata* (Figure 5). The thickness of the spongy mesophyll cells changed significantly (*p* < 0.001) in water-deficit stressed *Q. serrata* seedlings. Specifically, the individual cells generally shrank by approximately 40–50% (Figure 4 and Figure 5).

In this study, the vein density and loopiness increased significantly (*p* < 0.05) in the WS-treated seedlings of *Q. serrata*, while a non-significant effect was observed in *Q. acutissima*, not only in vein density, but also in the other venation traits measured (Table 1). The vein distance was significantly lower in the WS-treated leaves of *Q. serrata* than in the WW-treated leaves by nearly 3 mm (Table 1).

**Figure 6:**
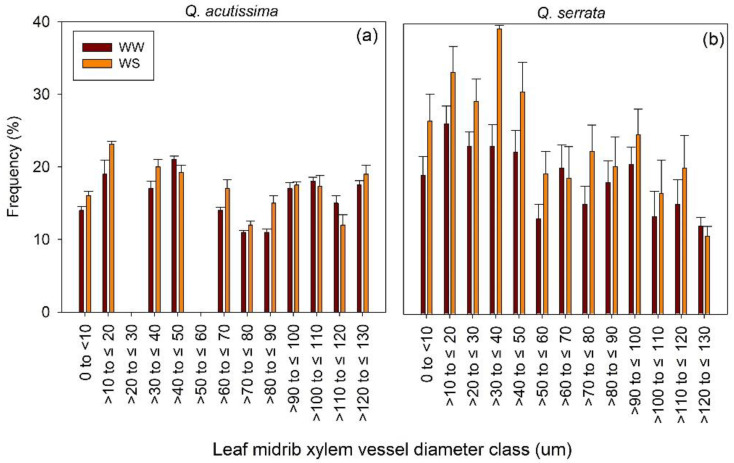
Frequency distribution of leaf midrib xylem vessel diameter classes of (**a**) *Quercus acutissima* and (**b**) *Q. serrata* in well-watered (WW) and water-deficit stress (WS) treatments after eight weeks. Vertical bars indicate the SE (n = 10). Still-green leaves were the ones harvested for leaf anatomical traits measurement.

The xylem vessel diameter of the leaf midrib did not vary significantly between WW and WS treatments in all diameter classes for *Q. acutissima* seedlings, but it did show significant variation in the case of *Q. serrata* (*p* < 0.003). Small-sized xylem vessels (i.e., 10–50 µm) were more frequent in WS-treated seedlings compared with those in the WW-treated seedlings of *Q. serrata* (Figure 6). The variation between treatments was generally similar for large-sized vessels (i.e., >50 µm).

### 2.2. Leaf Physiological Traits in Well-Watered and Water-Stressed Seedlings

**Figure 7:**
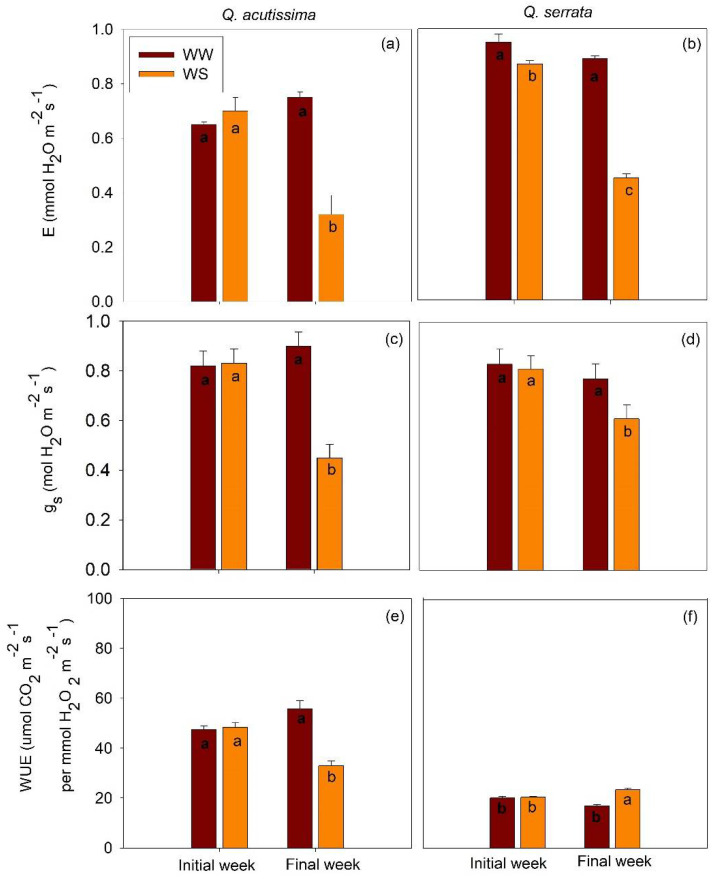
(**a**,**b**) Transpiration (E), (**c**,**d**) stomatal conductance (g_s_), and (**e**,**f**) water use efficiency (WUE) of *Quercus acutissima* and *Q. serrata* in well-watered (WW) and water-deficit stress (WS) treatments. Vertical bars indicate the SE (n = 10). Comparison means derived using Tukey’s test (*p* < 0.05) are shown for the significant interaction between water stress and time. Different lowercase letters indicate significant differences. Gas exchange measurements were performed in still-green leaves.

For the physiological traits, we found a significant effect (*p* < 0.01) of treatment × time interaction on leaf transpiration (E), stomatal conductance (g_s_), and water use efficiency (WUE) for both species. Generally, all these physiological traits declined significantly in WS-treated seedlings compared with WW-treated samples in the final week (Figure 7).

### 2.3. Principal Component Analysis Biplot

**Figure 8:**
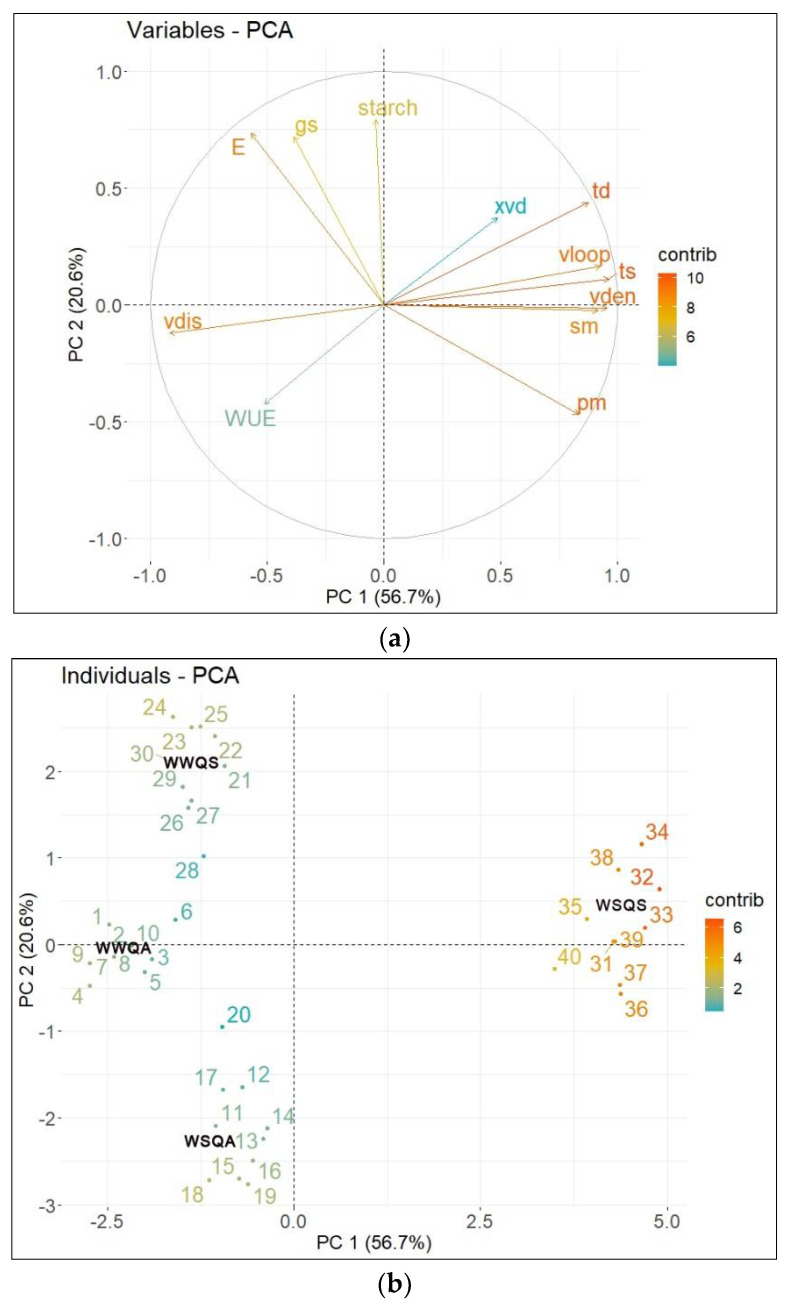
(**a**) Loading plot of the relationship between anatomical and physiological traits and (**b**) loading plot of individuals of well-watered (WW) and water-stressed seedlings of *Quercus acutissima* and *Q. serrata*. Abbreviations: WWQA—well-watered seedlings of *Q. acutissima* (samples 1–10)*;* WSQA—water-stressed seedlings of *Q. acutissima* (samples 11–20)*;* WWQS—well-watered seedlings of *Q. serrata* (samples 21–30); WSQS—well-stressed seedlings of *Q. serrata* (samples 31–40); gs—stomatal conductance; E—transpiration; vdis—vein distance; vden—vein density; vloop—vein loopiness; WUE—water use efficiency; pm—palisade mesophyll; sm—spongy mesophyll; ts—trichome-to-stomata ratio; td—trichome density; and xvd—xylem vessel diameter.

All the anatomical and physiological parameters were used in the principal component analysis (PCA) (Figure 8). Through PCA, the magnitude of the influence of water-deficit stress treatment on the measured parameters in *Q. acutissima* and *Q. serrata* and the correlations between variables could be observed. The PCA revealed that the first two components accounted for 77.3% of the variation in the dataset (Supplementary Materials, Table S1). Specifically, PC1 accounted for 56.7% of the variation and was highly related to trichome density, vein loopiness, trichome-to-stomata ratio, vein density, vein distance, palisade mesophyll thickness, and spongy mesophyll thickness. These variables showed a high association with water-stressed plants of *Q. serrata* (WSQS). PC 2 accounted for 20.6% of the variation and was highly correlated with starch content, xylem vessel diameter, g_s_, and WUE. The starch content and all the physiological traits are highly associated with all the well-watered seedlings of both species. The PCA plot also shows strong correlations between trichome density, vein loopiness, trichome-to-stomata ratio, vein density, vein distance, palisade mesophyll thickness, and spongy mesophyll thickness (Supplementary Materials, Table S2). The physiological traits were moderately to strongly negatively correlated with the anatomical traits measured (Supplementary Materials, Table S2).

### 2.4. Starch Content in Well-Watered and Water-Stressed Seedlings

**Figure 9:**
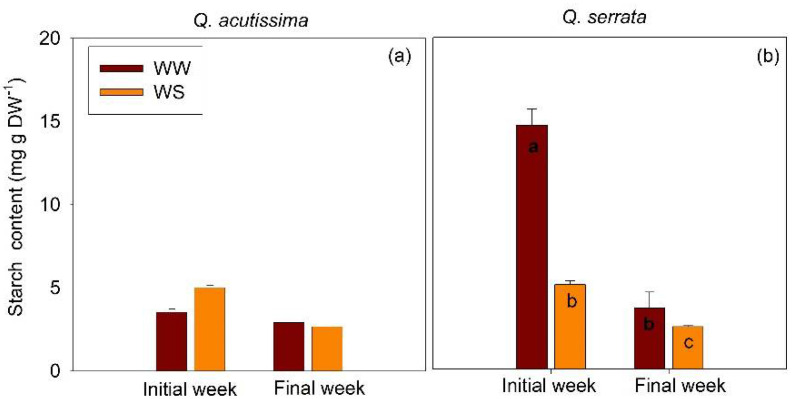
Starch content of (**a**) *Quercus acutissima* and (**b**) *Q. serrata* in well-watered (WW) and water-stressed (WS) treatments. Vertical bars indicate the SE (n = 8). Comparison means derived using Tukey’s test (*p* < 0.05) are shown for the significant interaction between water stress and time. Different lowercase letters indicate significant differences.

The treatments had no effect on the starch content in the stem of *Q. acutissima* in both experimental periods (Figure 9). A different pattern was observed in the case of *Q. serrata*, i.e., the starch content declined significantly (*p* < 0.03) in WS-treated seedlings in the initial week, and the values tended to decline further in the final week.

## 3. Discussion

As per our hypothesis, water deficit-treated seedlings exhibited more pronounced changes in anatomical and physiological traits compared with the well-watered samples, although these changes were more evident in *Q. serrata.* We revealed that the trichome types varied with progressive water-deficit stress in *Q. serrata*, but not in *Q. acutissima*. The density of non-glandular trichomes of *Q. serrata* increased with stress duration, suggesting that the species had gradually become more scabrous or hairy at the final week. The result agrees with the findings of Mediavilla et al. [27], who reported that some Mediterranean oak species exhibit a higher density of trichomes, which are indicative of water stress tolerance. Our results can be attributed to the need for the WS-treated seedlings to conserve water or avoid excessive water loss as the intensity of the stress increased. Dense non-glandular trichomes in the final week may have provided *Q. serrata* seedlings with adaptive and protective advantages necessary for the survival and maintenance of leaf greenness (Supplementary Materials, Figure S1). Our result is consistent with the findings of Gonzales et al. [28], who concluded that plants must be exposed to a degree of abiotic stress (e.g., drought and mechanical damage) to induce the production of non-glandular trichomes. The presence of a dense layer of leaf trichomes is a common characteristic of plants growing in dry areas [15,29,30]. Such a characteristic is thought to limit transpiration water loss by increasing leaf-air boundary layer resistance [31]. This explains the significantly lower E in the WS-treated group at the final week of the experiment and the negative correlation between E and trichome density of *Q. serrata* seedlings based on the PCA plot. Similarly, Lenssen et al. [32] reported a significant and negative correlation between the drying rate and the density of total trichomes in the *Medicago* species. Our results indicate that the increased non-glandular trichomes density may help reduce water loss via transpiration. This is supported by the significantly higher trichome-to-stomata ratio (T/S) in the WS compared to WW treatments in *Q. serrata*, particularly in the final week. Stomata have a key role in transpiration; hence, a higher TS in *Q. serrata* could result in a higher water use efficiency. Further, the decrease in glandular trichomes in the final week can be attributed to the stress-induced changes in morphological characteristics as the soil moisture decreased in a way that would invest more resources into the production of unicellular non-glandular hairs or nonsecretory trichomes. This is because glandular trichomes have multicellular glandular heads, which secrete secondary metabolites that may require more resources during the development or biosynthesis of the secondary metabolites compared with the nonsecretory trichomes. However, water-stressed *Q. acutissima* seedlings showed an even lower E, while not displaying higher trichome density than well-watered plants. PCA result showed that WW-treated seedlings of *Q. acutissima* were strongly associated with vein distance, which did not vary between WW and WS. The vein distance of *Q. acutissima* is higher than that of *Q. serrata* in both WW and WS, indicating that the former species has lower minor veins than the latter species. Minor veins are responsible for the bulk distribution of water during transpiration [33]. Thus, higher vein distance (i.e., the lower density of minor veins) may have resulted in even lower transpirational water loss in *Q. acutissima*. This suggests that E in *Q. serrata* could have been much higher because of the lower vein distance, if not for the increase in trichome density, although further investigation is needed to verify this.

Here, we found a moderately negative correlation between g_S_ and trichome density, and this is consistent with the PCA results of Shahzad et al. [34], who concluded that trichome density decreased with g_S_ and photosynthesis rate. The results of the present study can be explained by the ability of trichomes to directly control gas fluxes and diffusion resistance in the leaves by increasing the leaf boundary layer, which can influence the transfer of CO_2_ in the leaf. An increased leaf boundary layer may have controlled carbon uptake via stomata, but may have also trigger transpirational water loss. The magnitude of the effects on the gas-exchange rates, however, may be influenced by other leaf traits, such as leaf size and stomata [35]. Thus, the higher T/S may have prevented detrimental transpirational water loss from happening in the case of *Q. serrata*, although there was a significant decrease in E in WS-treated seedlings.

The results of the present study showed that the mesophyll structure, including thickness, compaction, and the ratio of the palisade to spongy mesophyll tissues, may be important structural components of a leaf that can explain the rates of CO_2_ conductance. We revealed that the WS-treaded seedlings had thicker palisade mesophylls than the WW-treated samples, in the leaves of both *Q. acutissima* and *Q. serrata*. Leaves with thicker palisade mesophyll could contain more chloroplasts [36], although we did not measure the chloroplast concentration in this study. However, the adjustment in the thickness of palisade mesophyll of *Q. acutissima* and *Q. serrata* seedlings may have increased the number of sites for CO_2_ assimilation per unit leaf area, resulting in normal photosynthetic activity, even if the g_s_ was declining due to water-deficit stress. This explains the observed higher survival rate despite lower g_s_ in the WS-treated seedlings of both species. Moreover, the individual cells of spongy mesophyll generally shrank in the WS-treated seedlings of *Q. serrata*. This further explains the observed lower g_s_ because a thinner spongy mesophyll could result in restricted entry and diffusion of CO_2_ in the leaf [37,38]. Ennajeh et al. [39] found a similar result, i.e., the thickness of the upper palisade of olive trees increased by 17% when subjected to water stress. A significantly thicker palisade was also observed in drought-resistant tropical trees when subjected to soil moisture deficit, and the photosynthesis rates correlated positively with the palisade mesophyll thickness [40].

The coordinated effects of thicker palisade mesophyll and specialized venation architecture can explain the observed higher WUE in *Q. serrata*, particularly in the final week. In this study, thicker palisade mesophyll may have resulted in a high WUE via improved resistance to water flow within the tissue, based on the observed strong positive correlation between palisade mesophyll and venation and/or vessel traits. We also found that the WUE was negatively correlated with palisade mesophyll and venation and/or vessel traits, suggesting that any changes in these traits would significantly influence WUE. Specifically, we found that the small-sized xylem vessels were more frequent in the WS-treated seedlings compared with the large-sized vessels observed at the final week, and these changes may have required the species to improve water flow resistance. If the small-sized vessels are embolized or damaged due to water-deficit stress, the effect on total water flow in the leaves would also be small [41,42]. Conversely, the large-sized vessels, when damaged, would result in a larger effect on total water flow compared with damage to small-sized vessels [41,43]. The transport of minerals and dissolved sugar to the cells of leaves is also influenced by the size of the vessels [44]. In addition, we found that the vein density and loopiness, which are strongly and positively correlated with palisade mesophyll, increased significantly in the WS-treated seedlings of *Q. serrata*. The vein distance, which showed strong negative correlation with palisade mesophyll, was also significantly lower in the WS-treated leaves of the species. The results suggest an improved vein network and efficiency of water transport and gas exchange within the leaf of *Q. serrata* under progressive water stress. Although high vein density and loopiness could imply high leaf carbon investment, some vein networks can serve as alternative routes for water movement in case of water deficit stress-induced damage.

In this study, the starch content declined significantly in the WS-treated seedlings of *Q. serrata* even as early as the final week of the experiment. Guo et al. [45] also reported a significant reduction in stem starch concentration by 69.8% under no irrigation treatment and only 39.1% under control. Our results can be ascribed to the bifunctional role of starch in carbon allocation in response to the environment. Starch plays a dual role in the carbon budget, acting as both a source for growth and development and as a sink for mechanical support [46]. Thus, such a decline may be due to the allocation or consumption of the starch reserves to the construction of mechanical strengthening tissues, such as the improved palisade mesophyll thickness, trichome density, venation architecture, and the number of small-sized xylem vessels in the leaves of the seedlings. These changes in the leaf anatomical structures were greatly needed in order to reduce water loss via transpiration and enhance water use efficiency, rather than increasing photosynthetic carbon gain as the water stress progressed. This consumption of starch reserves was demonstrated in the continued decline in starch concentration in the final week of the experiment. The results can also be associated with the observed lower g_s_ under water stress, resulting in constrained carbon assimilation in the leaves of *Q. serrata* seedlings. This could explain the observed negative correlation between starch content and all of the physiological traits. The water stress may have induced stomatal closure, which further limited the carbon gain. A limited supply of carbon could not provide the carbon demand of the seedlings for growth and metabolism as the water stress progressed. Hence, *Q. serrata* seedlings may have largely relied on the starch reserves to continue to survive and fuel metabolism. Moreover, starch is converted to soluble sugars to help maintain the leaf water content and regulate osmotic adjustments under drought stress [47,48]. In our previous work, we found that the concentration of total soluble sugar significantly decreased in the WS-treated seedlings of *Q. acutissima* and *Q. serrata* [49]. This further explains the decline in starch reserves in the WS-treated seedlings of *Q. serrata* in the present study.

## 4. Materials and Methods

### 4.1. Study Site

The experiment was conducted from May to September 2020 in a greenhouse at Chungnam National University (36°22′12″N, 127°21′17″E) located in Yuseong-gu, Daejeon, Korea. The mean monthly air temperature was 24.56 °C and the relative humidity was 75.28% (Supplementary Materials, Figure S2). The climatic data was obtained using the Onset HOBO air temperature sensor (Optic USB Base Station, U23 Pro v2, MA, USA). A knitted shade-cloth was installed on the roof of the greenhouse to regulate the temperature inside the greenhouse, since the experiment was conducted in the summer season. During rainy days, the windows of the greenhouse were closed to avoid rain splash. Destructive insects and weeds were controlled using a pesticide and a weedicide, which were sprayed on the leaves once a month.

### 4.2. Plant Materials and Experimental Design

Containerized seedlings (c.a., 1 year old) of fast-growing *Quercus acutissima* Carruth. and *Quercus serrata* Murray were used for this study (Supplementary Materials, Figure S3). The seedlings had initial stem diameter (SD) and height growth of 5.34–7.52 mm and 0.45–0.64 m, respectively. Seedlings were sourced from well-managed commercial nurseries in Korea and were planted in a 450-L pot. Each pot was filled with artificial soil, which was loamy sand in texture, with 7.01 pH, 7.28 cmol kg^−1^ CEC, and 1.29% soil organic matter (SOM). No fertilization application was made during the duration of the experiment.

The pots were organized randomly in the greenhouse, with ten replicates for each treatment and species. Each pot was planted with ten seedlings, following an approximately 10-cm distance between seedlings. Dead and inferior seedlings were replaced with good ones several weeks after planting. Before the water stress treatments were imposed, we subjected the seedlings to a 5-month acclimatization period to grow roots and adjust to the greenhouse environmental conditions. A rhizobox was inserted into selected pots (in the middle of the greenhouse) to monitor the production of fine roots (Supplementary Materials, Figure S4) to ensure that all the seedlings were ready for water-deficit stress imposition. During the five months, we watered the seedlings every two to three days until the first day of the treatment imposition, using an automatic irrigation system.

**Figure 10:**
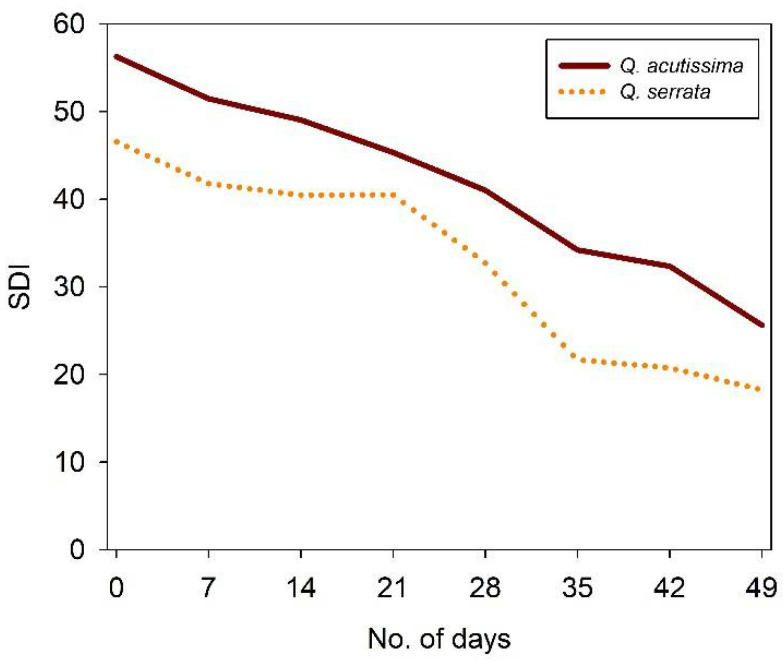
Average weekly substrate dryness index (SDI) of all pots of *Quercus acutissima* and *Quercus serrata* from day 0 (no treatment) to day 49 of treatment imposition.

In this experiment, we subjected the seedlings to two watering treatments: well-watered (WW, control) and water-deficit stress (WS). This was done when seedlings had already shown evidence of new root growth. The soil volumetric water content (VWC in %) was regularly observed by frequency domain technology (70 MHz, FDT) using 5 cm long probes (ECH2O EC- 5, n = 4) vertically inserted into the pot. The mean daily readings of VWC were logged at a 5 min interval during the experiment using a data logger (ZL6, Meter Group Inc., United States) via the ZENTRA Cloud (Meter Group Inc., United States). Here, the VWC under the WW treatment was sustained at 40–45% (field capacity of the soil after 2–3 days). Contrastingly, the VWC at WS treatment was allowed to drop gradually from 40 to only 8% (permanent soil wilting point), following modified procedures outlined by Jimenez et al. [50] and the theoretical basis of the water-deficient stress experiment [51]. Moreover, the substrate dryness index (SDI) was computed as the ratio of VWC to transpiration rates for each species to quantify the level of stress that they were suffering throughout the experimental period (Figure 10). Here, the lower the SDI value, the more serious the water stress.

### 4.3. Leaf Anatomical Traits Measurement

On the first (initial) and eighth (final) weeks of the experiment, ten fully expanded and healthy leaves attached to an orthotropic branch were randomly collected from each pot and species for the measurement of anatomical traits. All leaves had similar sun exposure and internodal positions on twigs and were collected consistently in the morning (i.e., 8:00 am to 10:00 am). The leaf development in the highest orthotropic branch was monitored before treatment application to ensure that leaves used were the ones produced only during the treatment period. During collection, leaf samples were sealed in plastic bags and temporarily stored in a cold storage box for further laboratory analysis.

The schematic diagram for the measurement of all leaf anatomical traits is presented in Figure 10. For trichome and stomatal density determination, epidermal leaf imprints were taken on the abaxial epidermis using the leaf epidermal impression technique and observed under a compound light microscope (Nikon Eclipse E200, Seoul, South Korea) equipped with LAS X imaging software (Leica Microsystems Ltd., Wetzlar, Germany). The trichome density (number mm^−2^) and stomatal density (number mm^−2^) were quantified in ImageJ [52]. The trichome-to-stomata ratio (T/S) was calculated as trichome density divided by stomatal density in a given leaf area.

**Figure 11:**
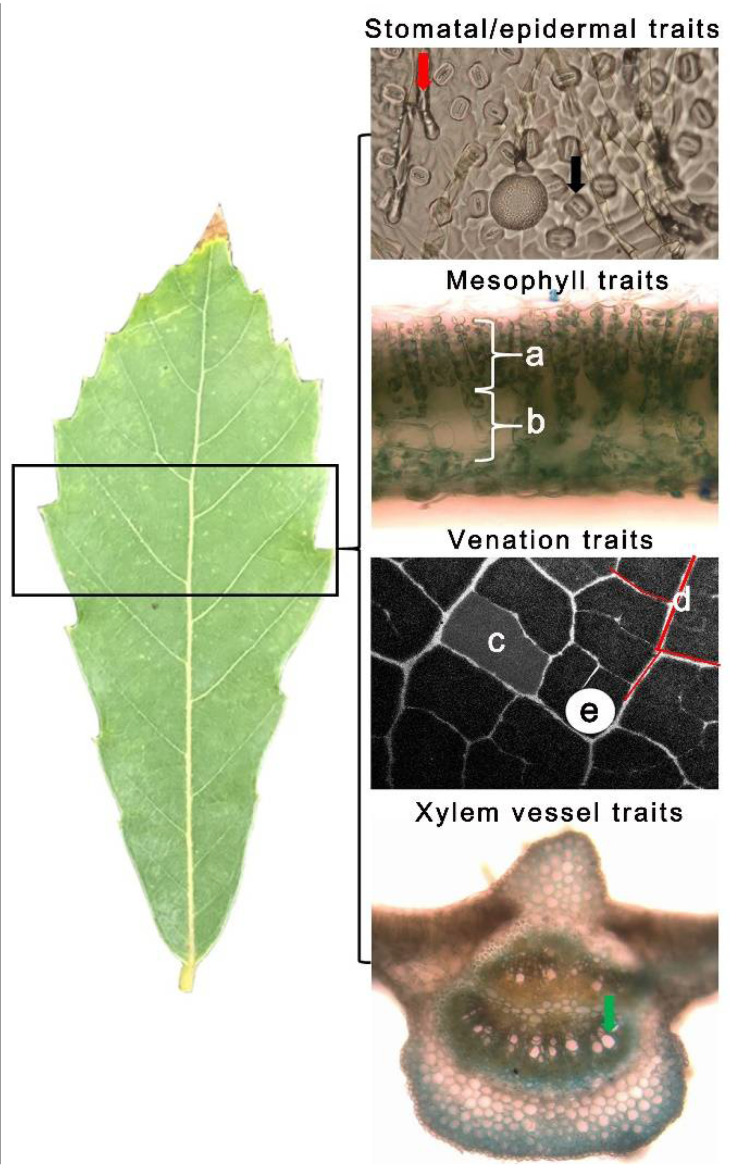
The schematic diagram for the measurement of the stomatal density (black arrow), trichome density (red arrow), mesophyll traits showing (a) palisade tissue and (b) spongy tissue, venation traits showing (c) loopiness, i.e., number of closed loops in the FOV divided by the FOV area, (d) vein density, i.e., the total length of veins in the FOV divided by the FOV area, (e) vein distance, i.e., the mean diameter of the largest circular masks that can fit in a closed loop, and xylem vessels (green arrow) [53] measured in the leaves of *Quercus acutissima* and *Q. serrata*.

Leaf samples (c.a., 1 mm × 2 mm) were cut from the middle portion of the leaves (Figure 11) and fixed in microcentrifuge tubes containing fixative solution at 4 °C for several weeks. Samples were dehydrated in a graded series of ethanol solutions (50, 65, 95, and 100%) at room temperature for one month. We used the freehand sectioning method following the modified procedures used by Hernandez et al. [49] to obtain very thin (c.a., 8–10 µm) cross-sections, which were then stained using Toluidine blue solution. The staining procedure was performed to easily determine the anatomical tissues of the leaf in the cross-section under a compound light microscope. The thickness (µm) of palisade and spongy mesophyll tissues and the diameter of all xylem vessels in the leaf midrib were measured using the same image processing software. The diameter of all xylem vessels in the leaf midrib was categorized using 10 μm diameter classes.

For the measurement of venation traits, ten leaves were collected from each treatment and species and were also subjected to a dehydration series using ethanol from 30% to 100% concentrations. We did not skeletonize the leaves because the midrib and second-and third-order veins were already visible after the last stage of the dehydration series. The vein density (mm mm^−2^), vein distance (mm), and vein loopiness (count mm^−2^) were measured in each leaf sample, as shown in Figure 11 [53].

In all microscopic examinations, five fields of view (FOV) at ×400 magnification were photographed from each sample. The values obtained from the FOVs were averaged for each leaf sample, and the average values of ten leaves were recorded for each species.

### 4.4. Leaf Gas Exchange Measurement

From each treatment and species, ten seedlings were selected for leaf gas exchange measurement, which was carried out between 9:00 am and 12:00 pm. Sun-exposed, healthy, and fully expanded leaves attached to an orthotropic branch (5th–6th nodes from the apical bud) of the seedlings were used in the study. The measurement was done only in still-green leaves as older leaves, which were produced before treatment imposition, were already starting to wilt as early as the 5th week of the experiment (Supplementary Materials, Figure S1). The stomatal conductance (g_s_, mol H_2_O m^−2^s^−1^) and transpiration (E, mmol H_2_O m^−2^s^−1^) were measured using a portable gas exchange measuring system (LI- 6400XT, LI-COR Inc., Lincoln, UK). The water use efficiency (WUE, μmol CO_2_m^−2^s^−1^ per mmol H_2_O m^−2^s^−1^) was also determined by dividing the photosynthesis rate by E. During the measurements, the saturating light was 1500 µmol m^−2^s^−1^, the leaf temperature was 26 °C, the CO_2_ concentration was 400 µmol mol^−1^, and the relative humidity was between 70 and 75%.

### 4.5. Starch Content Determination

In this study, we harvested eight seedlings from each treatment and species for the analysis of the starch content at the initial and final weeks. The harvesting was performed at dawn, when the seedlings were not photosynthetically active. An approximately 5 cm length of stem sample was cut from the base of each seedling, covered with wet tissue, placed in a cold storage container while in transport (0.5–1 km), and stored immediately in a −21 °C laboratory freezer until further analysis. Stems were microwaved for 2–3 min at 500 W to stop the enzymatic activities, and debarked by removing only the outer layer of the stem using a razor blade. Thereafter, the stems were then oven-dried at 60 °C overnight and ground to produce 50 mg of fine powder with a ball mill. The powder was suspended in 100 mL of 80% EtOH during extraction, immersed in the water bath at 90 °C for 10 min, and centrifuged for 10 min at 3000 rpm. After removing the supernatant with the use of GF/C filter paper, the pellet was dried overnight to eliminate residual ethanol. We followed the classic protocol for the rapid quantitative determination of carbohydrates with Dreywood’s Anthrone Reagent [54]. The reagent was prepared by dissolving 200 mg of anthrone in 100 mL concentrated H_2_SO_4_. Standard glucose solutions (200, 400, 600, 800, 1000 µg mL^−1^) were prepared in tubes, to which 4 mL of anthrone solution was added. The tubes were heated in a water bath for 10 min, cooled to room temperature, and then topped with 5 mL distilled water. Starch was solubilized by sonication in dimethyl sulfoxide (DMSO). The concentrations (mg g^−1^ DW) of starch (measured as glucose equivalents) were read at 620 nm by spectrophotometry (UVmini-1240, Shimadzu, Japan) after the phenol–sulphuric acid reaction. A calibration curve (Supplementary Materials, Figure S5) was made to calculate the equivalent weights of the unknown samples. The starch content was calculated by multiplying the obtained values of glucose by 0.9.

### 4.6. Statistical Analysis

The normal distribution of the data collected was first evaluated using the “Shapiro.test” package in R statistical software (version R-3.5.1). Data were subjected to a one-way ANOVA to determine the effects of the treatment on the thickness of palisade and spongy mesophyll tissues and the diameter of xylem vessels. Two-way ANOVA was also used to test for significant differences in trichome density, trichome-to-stomata ratio, venation traits, all physiological traits, and starch content between treatments within time points (i.e., initial and final weeks). Means were compared using Tukey’s HSD post hoc test (α = 0.05). The relationships between physiological traits and anatomical traits measured from well-watered and water-deficit stressed seedlings were determined using principal component analysis (PCA). Only the principal components (PCs) with eigenvalues greater than one were used in the construction of the PCA plot (Supplementary Materials, Table S1). All calculations were performed in R software at a significance level of α = 0.05.

## 5. Conclusions

The present study demonstrated the effects of water-deficit stress on morpho-anatomical traits, physiological traits, and starch content in *Q. acutissima* and *Q. serrata* seedlings. We revealed that, in the water stress-treated plants, the stomatal and epidermal traits, mesophyll traits, venation traits, number of small-sized xylem vessels, and the stem starch content—in order to cope with the increasing effects of progressive water stress on transpiration rate, stomatal conductance, and water use efficiency—were all significantly altered. Between the two oak species, the adaptive traits related to water stress seemed to be more evident in *Q. serrata* compared with *Q. acutissima*. Thus, the coordinated modifications in leaf morpho-anatomical traits, physiological traits, and stem starch content play an important role in the survival of *Q. acutissima* and *Q. serrata* seedlings in water-deficit stress environments, although *Q. serrata* may have higher potential to survive prolonged water stress than *Q. acutissima.*

## Figures and Tables

**Table 1:** Effects of well-watered (WW) and water-deficit stress (WS) on vein density, distance, and loopiness of *Quercus acutissima* and *Q. serrata* after eight weeks. Different lowercase letters indicate significant differences between treatments at α = 0.05. Values in parenthesis are the standard errors (n = 10). Still-green leaves were the ones harvested for leaf anatomical traits measurement.

**Species**	**Treatment**	**Vein Density** **(mm mm^−2^)**	**Vein Distance** **(mm)**	**Vein Loopiness** **(Count mm^−2^)**
*Q. acutissima*	WW	5.93 (0.29)	8.43 (0.20)	8.61(0.43)
	WS	6.18 (0.23)	8.43 (0.15)	9.32 (0.21)
*Q. serrata*	WW	5.47 (0.27) ^b^	8.06 (0.28) ^a^	9.82 (0.59) ^b^
	WS	12.73 (0.53) ^a^	5.15 (0.17) ^b^	13.74 (0.54) ^a^

## Data Availability

All the data used are already reflected in the article. Other relevant data may be available upon request from the authors.

## Supplementary Materials

The supporting information can be downloaded at: https://www.mdpi.com/article/10.3390/ijms23158640/s1.
