# Supplementary material for: The Leaf Trichome, Venation, and Mesophyll Structural Traits Play Important Roles in the Physiological Responses of Oak Seedlings to Water-Deficit Stress
Source: Int J Mol Sci. 2022 Aug 3;23(15):8640. doi: 10.3390/ijms23158640 (PMC9369340; doi:10.3390/ijms23158640)
Supplement: Supplementary file 1 [file ijms-23-08640-s001.zip › ijms-1840277-supplementary.pdf]

Supplementary Materials

Table S1. Eigenvalues of the twelve components of the principal component analysis loading plot.

Components	Eigenvalue	Variance percent (%)	Cumulative variance percent (%)
**PC1**	6.81	56.74	56.74
**PC2**	2.47	20.57	77.31
**PC3**	0.95	9.41	86.72
**PC4**	0.88	5.79	92.50
**PC5**	0.45	3.75	96.26
**PC6**	0.13	1.10	97.35
**PC7**	0.11	0.94	98.30
**PC8**	0.09	0.78	99.08
**PC9**	0.04	0.37	99.45
**PC10**	0.03	0.27	99.72
**PC11**	0.02	0.19	99.91
**PC12**	0.01	0.09	100.00

Table S2. Contribution of the variables to the first two components/dimensions of the principal component analysis loading plot and the correlation between variables.

	PC1	PC2
**td**	-0.96	0.85
**ts**	-0.96	0.72
**pm**	-0.70	0.11
**sm**	-0.88	0.47
**vden**	-0.92	0.53
**vdis**	0.92	-0.62
**vloop**	-0.93	0.69
**xvd**	-0.53	0.59
**E**	0.37	0.12
**gs**	0.21	0.10
**WUE**	0.61	-0.86
**starch**	-0.13	0.51

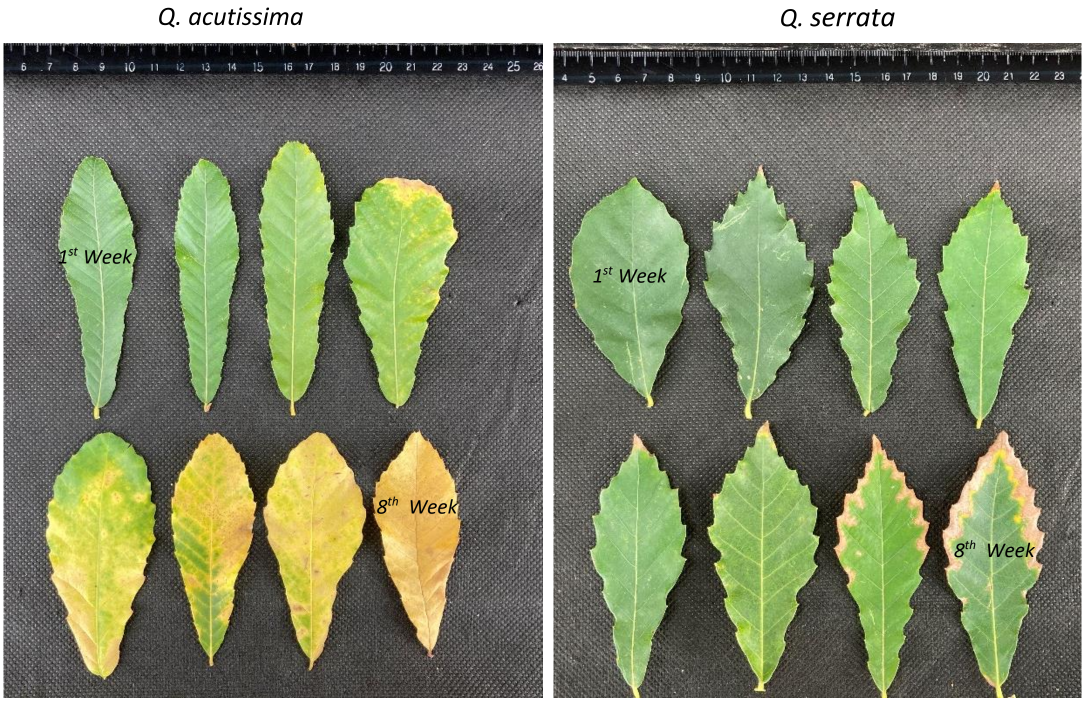

Figure S1. Greenness of *Q. acutissima* and *Q. serrata* from the 1st week until 8th week of the experiment.

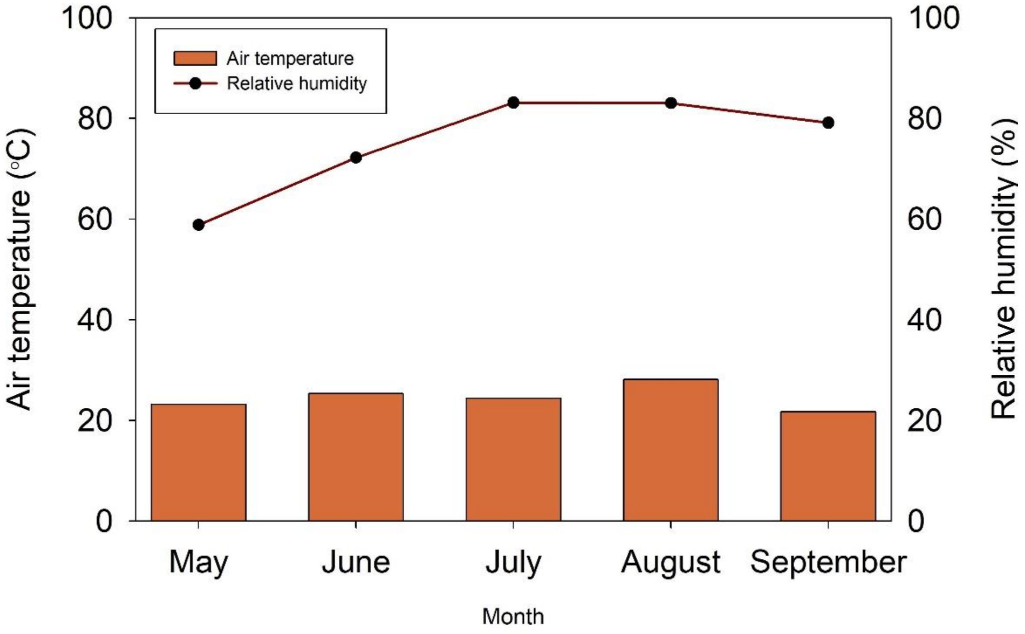

Figure S2. Mean monthly air temperature and relative humidity in the greenhouse throughout the experimental period.

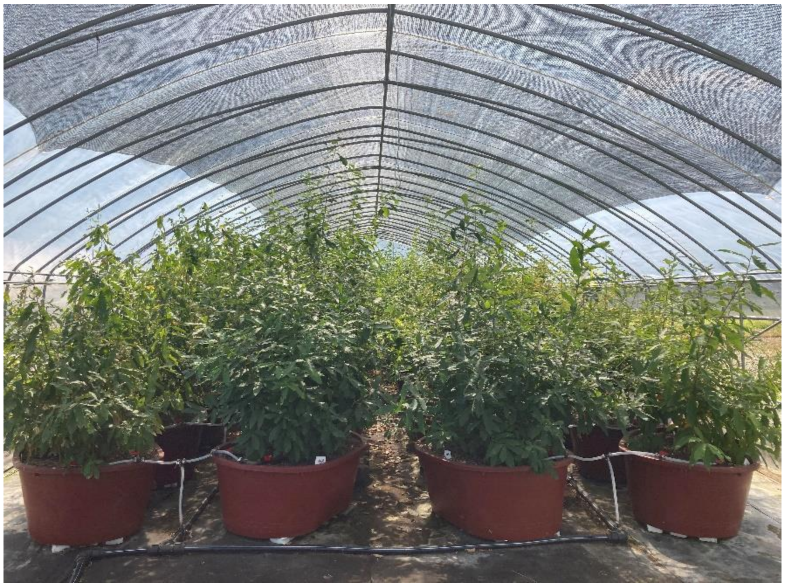

Figure S3. Experimental setup of the present study.

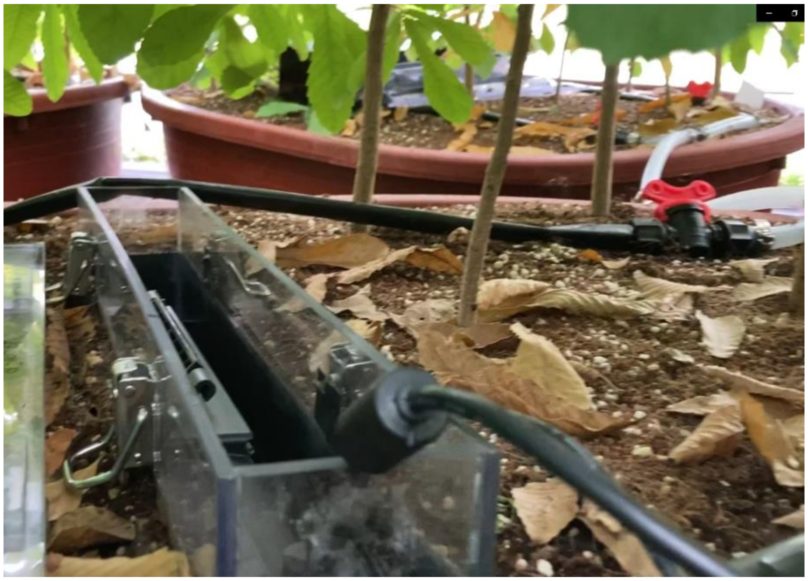

Figure S4. Rhizobox and flatbed scanner used in the present study to monitor root growth.

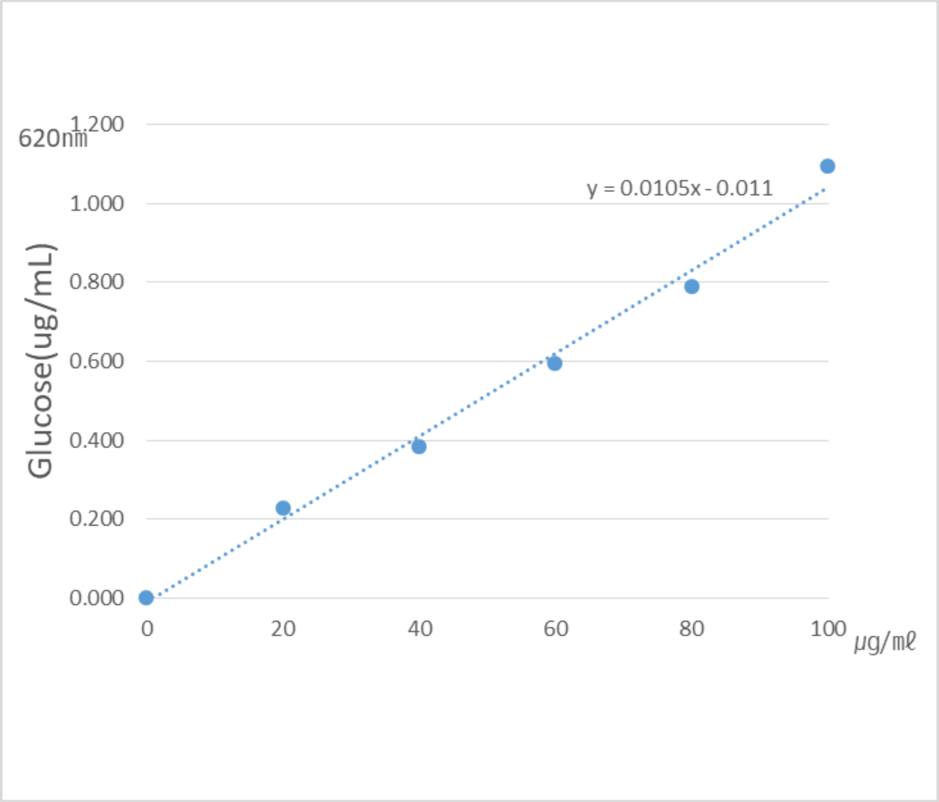

Figure S5. A calibration curve made to calculate the equivalent weights of the unknown concentration of starch from the stem samples.
